# Enhancing Future Health Equity Plans: Insights from an Evaluation of Public Health Equity Resources in Oregon

**DOI:** 10.1089/heq.2023.0172

**Published:** 2024-01-09

**Authors:** Alyssa Pinoliar, Genevieve Ellis, Marie Boman-Davis, Erika Westling

**Affiliations:** ^1^ ORI Community and Evaluation Services, Springfield, Oregon, USA.; ^2^ Public Health Division, Department of Health and Human Services, Washington County, Oregon, USA.; ^3^ Oregon Research Institute, Springfield, Oregon, USA.

**Keywords:** public health, health equity, health equity action plan, public health modernization, local public health authority, Oregon, health and human services

## Abstract

Health Equity Action Plans (HEAPs) are a recent strategy employed across health and human services to promote health equity. To inform the development of future HEAPs, as well as to build upon previous initiatives, we evaluated 52 health equity plans and resources from Oregon counties using five criteria: creation date, process orientation, racial equity lens, metrics, and community engagement. When developing future HEAPs, we recommend explicit commitments to collaborate with marginalized communities, to establish measurable goals and defined metrics for assessing progress, to include voices and perspectives of those affected by health inequities, and to detail community strengths, assets, and resources.

## Introduction

Oregon state defines health equity as “all people can reach their full health potential and well-being and are not disadvantaged by their race, ethnicity, language, disability, age, gender, gender identity, sexual orientation, social class, intersections among these communities or identities, or other socially determined circumstances,” and aims to create a health system that ends health inequities by 2030.^1^ The COVID-19 pandemic brought renewed attention to preexisting health inequities worldwide.

In the United States, Health Equity Action Plans (HEAPs) are a recent strategy employed across governmental health and human services to promote health equity.^2^ Local public health authorities are required to create a HEAP to work toward achieving this goal.^3,4^ However, little guidance is available regarding what constitutes a HEAP, although one recent article summarizes how states in the Midwest are promoting health equity.^5^ We systematically reviewed what had been done in the state of Oregon thus far, to identify potential plan features to include in a local public health authority HEAP. Here, we provide our insights from this review, to inform the development of future policies and plans designed to advance health equity.

## Public Health Modernization

Oregon's Public Health Advisory Board developed a phased plan to modernize the state's public health system over a period of 6 to 10 years, using funds allocated for this purpose.^4^ The first phase of implementation prioritizes enhancing the state's ability to advance health equity and cultural responsiveness.^4^

## Health Equity Action Plans

In guiding documents provided by the Oregon Health Authority, the HEAP is described as an “action plan that addresses key findings from the internal assessment and includes organizational changes that support a health equity lens and cultural responsiveness.”^4^ Additionally, it states that the action plan “includes metrics and an accountability structure that identifies responsible work units, tasks, timelines and performance measures.”^4^

## Oregon Public Health Equity Resources

To determine if existing resources exist in Oregon counties that could inform the development of a local public health authority HEAP, we began with a broad online search, using terms such as “Oregon Health Equity Action Plan.” Next, we went to each of the 36 Oregon county public health division webpages and searched for “Health Equity Action Plan,” “Health Equity,” “Community Health Improvement Plan,” “Community Health Needs Assessment,” “Community Health Assessment,” “Regional Health Improvement Plan,” “Regional Needs Assessment,” and “Strategic Plan.” Via these searches, we documented 52 potentially relevant health equity plans and resources (Table 1). Resources included statements, reports, and assessments.

**Table 1.** Oregon County Health Equity Resources

Type of resource	No. of resources	Oregon county/tribe	Resource
Health Equity Plan	1	**Columbia River Gorge**	**Health Equity Plan 2020^6^**
Health Equity-Centered Resources	6	Baker	COVID-19 Health Equity Plan (2021)^7^
		Clackamas	Health Equity Framework 2022^8^
		Coos	Reproductive Health Equity Act 2023^9^
		Lane	Health Equity Report 2020^10^
		Marion	Equity Plan COVID-19 Update 8/31/21^11^
		Umatilla	Equity & Modernization 2023^12^
CHA; CHNA; CNA; RHA	24	Baker	2022 CHNA Update^13^
		**Clackamas-Multnomah- Washington**	**Healthy Columbia Willamette Collaborative CHNA 2022^14^**
		Crook-Deschutes- Jefferson-Northern Klamath-Confederated Tribes of Warm Springs	2019 Central Oregon RHA^a^^,^^15^
		Curry	CHA 2018^a^^,^^16^
		Douglas	CHA 2018^a^^,^^17^
		Gilliam	CHA 2019^a^^,^^18^
		Grant	CHA 2019^a^^,^^19^
		**Harney**	**2022 Harney County CHNA;^20^ Progress Report 2023^21^**
		Hood River	CHA 2019^a^^,^^22^
		Jackson-Josephine	2018 CHA^a^^,^^23^
		**Klamath**	**CHA 2021^24^**
		Lake	CNA 2020–2021^25^
		Lane	CHA 2019^a^^,^^26^
		Linn-Benton-Lincoln	RHA 2022–2026^27^
		Marion-Polk	CHA 2021 Update^28^
		Morrow	CHA 2021^29^
		Sherman	CHA 2019^a^^,^^30^
		**Statewide**	**2020–2024 Healthier Together Oregon State HIP^31^**
		Umatilla	CHA 2018^a^^,^^32^
		Union	2021 CHA^33^
		Wallowa	2022 CHNA^34^
		Wheeler	CHA 2019^a^^,^^35^
		**Yamhill**	**CHA 2022^36^**
CHIP, CHP; HIP; Regional Health Improvement Plans	14	Baker	CHP 2021^37^
		Benton	CHIP 2018–2022^a^^,^^38^
		Clatsop	CHIP 2013^a^^,^^39^
		**Columbia River Gorge**	**Regional CHIP 2020–2023^40^**
		**Crook-Deschutes- Jefferson-Northern Klamath-Confederated Tribes of Warm Springs**	**2020–2024 Central Oregon Regional Health Improvement Plan^41^/2022 Progress Report^42^**
		Curry	CHIP 2019^a^^,^^43^
		Douglas	CHIP 2019^a^^,^^44^
		Klamath	CHIP 2019^a^^,^^45^
		Lane	CHP 2021–2025^46^
		Lincoln	CHIP 2019–2024^a^^,^^47^
		Marion-Polk	CHIP 2020–2025^48^
		Morrow	HIP 2020^49^
		Wallowa	CHP 2021^50^
		Washington	CHIP 2020–2023^51^
		Yamhill	CHIP 2018–2022^a^^,^^52^
Strategic Plans	5	**Clackamas**	**Strategic Plan 2022–2024^53^**
		Crook	Strategic Plan 2016–2020^a^^,^^54^
		Deschutes	Strategic Plan 2016–2022^a^^,^^55^
		Linn	2012–2015 Public Health Strategic Plan, Updated 2016^a^^,^^56^
		Washington	Strategic Plan 2020^57^

Bold indicates a plan or resource that met all five criteria.

^a^ A plan or resource that was created before 2020.

CHA, Community Health Assessments; CHIP, Community Health Improvement Plans; CHNA, Community Health Needs Assessments; CHP, Community Health Plans; CNA, County Needs Assessment; HIP, Health Improvement Plan; RHA, Regional Health Assessment.

## Criteria for Evaluating the Health Equity Resources

To evaluate public health equity resources in Oregon counties, we established five criteria (Table 2). Our focus was on recently implemented resources, so the first requirement was that the resource was created in 2020 or later. If this criterion was met, we proceeded to evaluate the next four criteria. The second criterion was to assess whether the resource had a process-focus, meaning it provided a detailed plan on how to achieve the resource's stated health equity goals. The third criterion was to determine if the resource had a racial equity perspective, rather than a more general equity approach. The fourth criterion evaluated whether tools and metrics were included to track progress toward stated objectives. Finally, we assessed whether robust community involvement was evident, which served as our fifth and final criterion.

**Table 2.** Criteria for Evaluating Oregon County Health Equity Resources

Criteria	Description
Recently implemented	Created during or after 2020.
Process-focused	Includes a focus on internal and external work the county or public health division will need to do to achieve identified health equity outcomes.
Racial equity lens	Includes a lens of “leading with race,” as well as a definition of health equity and a description of included populations.
Tools or metrics	Includes accountability metrics to evaluate success; outlines strategies to be applied to ensure that equity practices in the plan are being followed.
Community involvement	Community partners or members were engaged in the planning process and their input was incorporated in the final plan.

## Evaluating Health Equity Resources by Criteria

Of the 52 total plans and resources, 21 were created before 2020, so were not subsequently evaluated on the remaining criteria. Of the 31 plans that were evaluated on the subsequent four criteria, 23 had a process-focus, 22 included a racial-equity lens, 22 specified tools or metrics to be used to assess progress, and 23 involved community voices. Eleven resources met all criteria. None were explicitly called a HEAP, but the Columbia River Gorge Health Equity Plan 2020^6^ came the closest to being a HEAP. The other 10 that met all criteria were more focused health equity resources (see bold plans in Table 1). This does not imply that counties throughout Oregon are not involved in this work, but it underscores the novelty of this initiative and the difficulty of integrating all these concepts into a single, comprehensive plan.

## Columbia River Gorge Health Equity Plan (2020)

As an illustration of our evaluation process, we will review the Columbia River Gorge Health Equity Plan (2020),^6^ the plan closest to a HEAP in Oregon. First, this plan satisfies our first criterion, as it was created in 2020. The plan has three overarching goals: (1) help agencies in the Gorge develop a better understanding and working knowledge of equity; (2) support agencies in assessing and understanding where each is on its journey toward equity; and (3) identify supports and resources to help these agencies adopt equitable policies and practices.

Second, this plan is process-focused, providing a detailed plan of action both internally and externally to achieve the stated goals. For instance, the plan outlines the development of guidance for community service providers and partners, which involve the county identifying and developing resources, tools, training, and services. The creation of these resources would help achieve plan goal three. External processes are also specified, such as ongoing facilitated meetings designed to assess the individual and collective strengths and needs of community partners, which would advance plan goal two.

Third, this plan adopts a racial-equity lens, identifying gaps between agencies and service providers to address equity issues, and specifically including Latinx and Native American serving organizations in the process. The plan also involves a process by which survey questions go through a community advisory council to ensure that the survey language is in plain English and Spanish. Surveys are also designed to be passed out or done in-person to ensure participation by those most impacted by health inequities. The Columbia River Gorge Health Equity Plan (2020) is available in Spanish as well.

Fourth, both internal and external assessment tools are provided. One internal tool is the Diversity, Equity, and Inclusion Spectrum Tool, which ensures accountability by assessing organizational progress toward health equity. Additionally, external data from a community health assessment is used throughout the plan to calculate an inequity measure, and updated metrics on this measure can be used to evaluate progress toward the three broad plan goals.

Finally, community involvement with this plan was robust and included collaborations with state, local, and regional partners. To develop the plan, three collaborative community health needs assessments were conducted, and quotes from individual community members were included in the plan. Six community partners were also highlighted.

## Recommendations

Based on our findings, we have five recommendations for local public health agencies embarking on creating a HEAP. Our first recommendation is to include a process-focused approach to achieving the specified health equity goals in the plan. While documents such as Community Health Improvement Plans often prioritize external metrics, they often lack sufficient detail on internal work plans. It is important to address this gap to ensure that staff have a clearly defined path forward for achieving the goals.

Second, we suggest making an explicit commitment to working with Black, Indigenous, and People of Color (BIPOC) communities, starting with a focus on race instead of broad equity statements. It is crucial to acknowledge the historical and current harm caused by local government and public health and demonstrate a clear commitment to engaging with BIPOC communities.

Third, include clear metrics that can be used to evaluate progress on the plan. Through this review, we saw examples of both progress- and outcome-metrics, both of which are necessary components of a HEAP. It will be particularly important to include community input in developing not only the metrics but also the strategies used to achieve health equity outcomes.

Fourth, we recommend intentionally including the voices of communities impacted by health inequities in the development of the plan. We saw this example clearly in the Columbia Gorge Health Equity Plan 2020^6^ and appreciated the clear attention to community engagement. We believe that including community voices will lead to a stronger planning process and development of programs that better meet the needs of impacted communities.

Finally, we recommend a focus on community strengths, assets, and resources. Often, there is a focus on needs, without the inclusion of strengths. Recognizing that communities may already have solutions in place can help build on strengths and identify where we can add funding, capacity, or other support to these efforts.

## Abbreviations Used

BIPOC: Black, Indigenous, and People of Color
CHA: Community Health Assessments
CHIP: Community Health Improvement Plans
CHNA: Community Health Needs Assessments
CHP: Community Health Plans
CNA: County Needs Assessment
HEAPs: Health Equity Action Plans
HIP: Health Improvement Plan
RHA: Regional Health Assessment
